# Data linkage of routinely collected electronic health records to characterise risk factors associated with antibiotic resistance in urinary isolates: an exemplar of NHS Scotland's Infection Intelligence Platform

**DOI:** 10.23889/ijpds.v1i1.294

**Published:** 2017-04-19

**Authors:** Eilidh Fletcher, Kim Kavanagh, William Malcolm, Camilla Wiuff, Nicholas Reid, Ashutosh Deshpande

**Affiliations:** NHS National Services Scotland; University of Strathclyde; Pharmacy department, NHS Ayrshire & Arran; Microbiology department, Royal Alexandra Hospital, NHS Greater Glasgow and Clyde

## Objectives

Urinary tract infections (UTIs) are amongst the most common infections treated in community and hospital settings. Initial antibiotic treatment of UTI is usually empirical, that is, where the prescriber has no definitive information on the organism or its antibiotic sensitivity.. Overall the prevalence of antimicrobial resistance is increasing, and specifically so for antibiotics commonly used for UTI.

## Approach

All positive urine samples included in the "Surveillance of Antimicrobial Resistance in Urinary Isolates in Scotland" dataset in the period from January 2012 to June 2015 (all NHS Health Boards in Scotland submit susceptibility data for up to eleven antibiotics on 400 positive urinary samples per quarter) were analysed. Cases were assigned a resistance status of Sensitive, Resistant or Multi-drug resistant based on the antibiotic susceptibility data recorded.

Using the NHS Scotland Infection Intelligence Platform all cases were linked to national coverage data: (i) hospital discharge data to create the Charlson score for comorbidity and (ii) patient-level community prescribing data to measure cumulative antibiotic exposure (number of defined daily doses) in the 3 months prior to infection. Risk factors associated with the infection susceptibility to antibiotics were assessed using multivariable multinomial logistic regression. 

## Results

40,984 positive urine samples were examined. Overall 29.0% were sensitive, 48.1% resistant and 22.9% multi drug resistant. Around a third of the cases (33.9%) had no antibiotic prescribing in the 3 months prior to infection. Age, care home residence and increasing comorbidity were both found to be associated with resistance and multidrug resistance. Cumulative antibiotic exposure had a clear dose-response effect. Those with 1-7DDDs were 1.2 times (95% CI: 1.11-1.29) more likely to have a multidrug resistant infection (compared to a sensitive infection) rising to 7.45 times (95% CI: 95% CI 6.45-8.6) for 29+ DDDs. Similar dose response held for resistant infection but at a lesser scale (1-7DDDs OR=1.36 (95% CI: 1.2-1.5) rising to OR=3.04 (95% CI: 2.38-3.89). 

## Conclusion

A clear effect of cumulative antibiotic exposure in the community and multidrug resistance in UTI cases has been demonstrated. Such quantification is key to ensuring and supporting robust antimicrobial stewardship policy and will form the evidence base for development of prescribing decision support tools for more patient centred treatment of UTI.

